# Iatrogenic Acute Ischemic Necrosis Due to Emergent Bleeding Control in Ventral Foramen Magnum Meningioma with Spinal Instability

**DOI:** 10.7759/cureus.7754

**Published:** 2020-04-21

**Authors:** Christ Ordookhanian, Ryan F Amidon, Talia Vartanian, Paul Kaloostian

**Affiliations:** 1 Medicine, University of California, Riverside, USA; 2 Neuroscience, University of California, Riverside, USA; 3 Physical Medicine and Rehabilitation, University of Southern California, Los Angeles, USA; 4 Neurological Surgery, Paul Kaloostian M.D. Inc., Riverside, USA; 5 Neurological Surgery, Riverside Community Hospital, Riverside, USA

**Keywords:** meningioma, acute ischemic necrosis, tinnitus, myelopathy, foramen magnum, aneurysmal clips, vertebral artery

## Abstract

Meningiomas are the most common benign intracranial tumors. They often require surgical resection and postoperative radiation/chemotherapy based on their histologic grade. While necrosis caused by preoperative embolization and spontaneous tumor infarction is appreciated by pathologists when staging meningiomas, intraoperative events including large bore artery occlusion may also alter the histopathologic picture of a benign meningioma. Hence, they should be considered when signs of unexpected ischemia and necrosis are found, as these same phenotypes are also hallmarks of a higher-grade disease. We describe a case of a man with a large ventral foramen magnum meningioma who underwent temporary intraoperative occlusion of the vertebral artery, leading to ischemic tumor necrosis with abundant neutrophil invasion when the tumor was eventually examined histologically.

## Introduction

Meningioma is an extremely common pathology, accounting for more than 30% of all primary intracranial neoplasms in the United States. It is more prevalent among older adults and women [1]. The incidence of meningiomas has nearly doubled within the last decade, mostly attributed to improved detection methodologies [1]. Peripheral vascular supplies are often prone to ischemic events and consequent necrosis, and this is typically observed after preoperative embolization or spontaneous infarction of meningiomas [2]. In our case, a foramen magnum meningioma was resected via a far lateral approach without preoperative embolization, ischemia, or infarction. After resection, there was evidence of necrosis and significant neutrophil presence characteristic of acute ischemia, produced by intraoperative events involving iatrogenic trauma, occlusion, and repair of the vertebral artery. This case uniquely demonstrates the presence of tumoral ischemia and necrosis resulting from intraoperative complications and the importance of taking such events into account when assessing the appropriate grade of a tumor and its postoperative treatment.

## Case presentation

**Figure 1:**
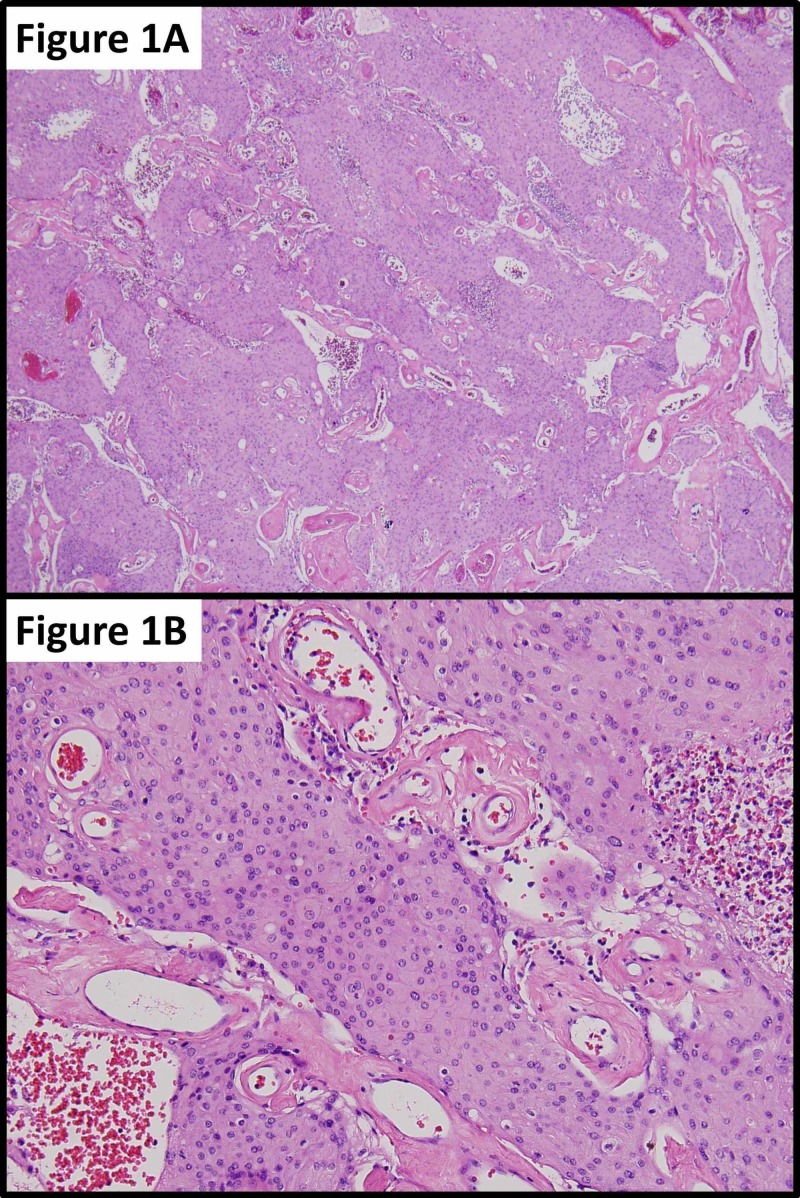
Meningioma showing scattered foci of ischemic necrosis (1A); magnified meningioma showing histological hallmarks of ischemic necrosis (1B) 1A: the histological examination of the tumor revealed a WHO grade 1 meningioma with foci of necrosis distributed throughout the sample; 1B: the magnified histological examination of WHO grade 1 meningioma with apparent pleomorphic nuclei, with high nucleus:cytoplasm (N/C) ratio and increased cellularity WHO: World Health Organization

**Figure 2:**
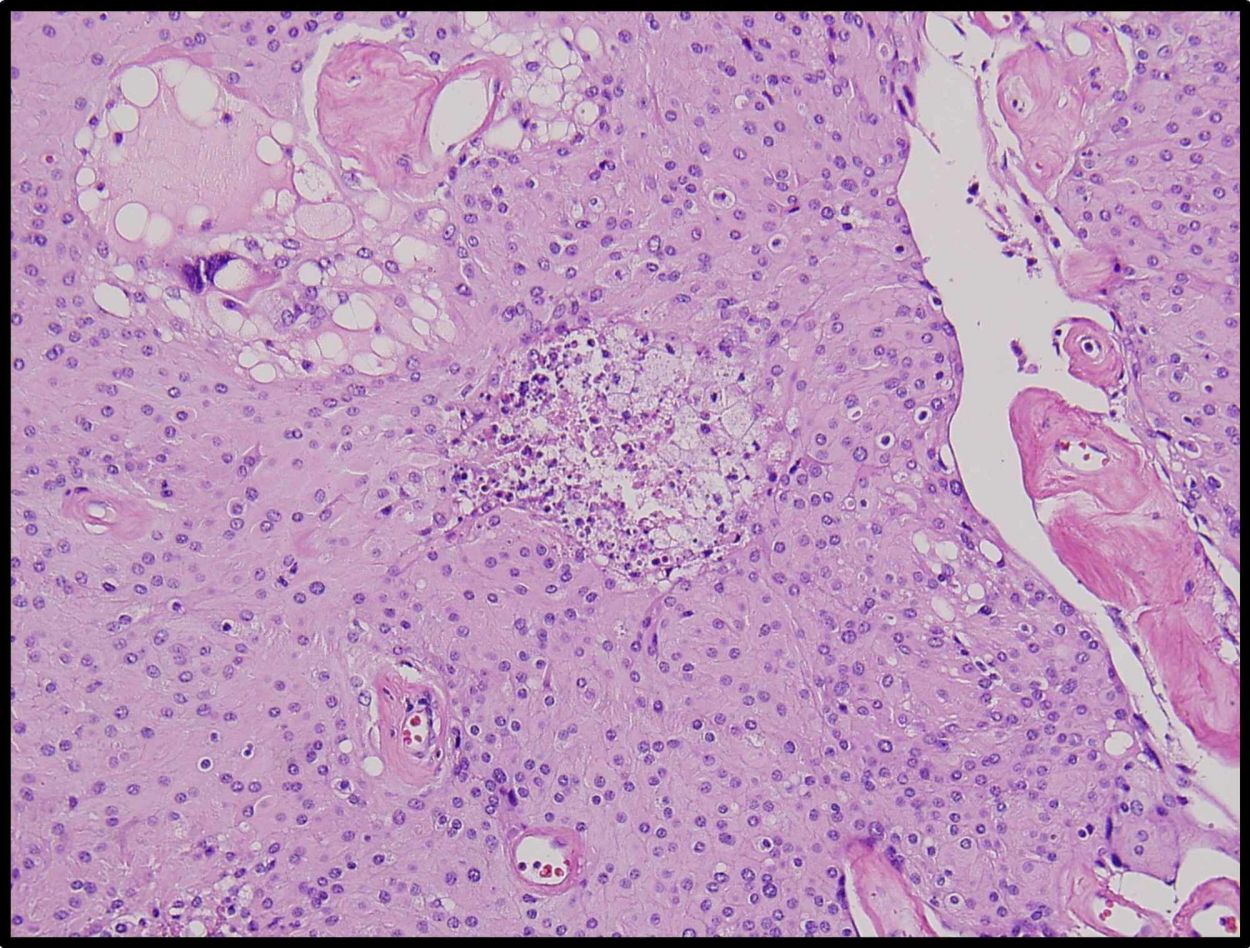
Meningioma showing scattered foci of ischemic necrosis showing neutrophil invasion Abundant neutrophil invasion was seen throughout the areas of necrosis in a pattern consistent with acute ischemic necrosis

A 46-year-old man with a history of a suboccipital craniectomy at age 12 for resection of posterior fossa astrocytoma, with subsequent radiation therapy, presented to our multidisciplinary brain tumor clinic with myelopathy, tinnitus, and hearing loss. Radiographic imaging of the craniospinal axis led to the diagnosis of a large skull-base foramen magnum C1-C2 tumor with significant spinal cord and brainstem compression. While undergoing extreme lateral transcondylar complex skull base approach for resection of the tumor, a large extradural branch, presumably to the tumor, was cauterized. The proximal branch was then inadvertently avulsed at its origin with the extradural left vertebral artery, which led to a high-flow arterial defect. Proximal and distal left vertebral artery blood flow was occluded with temporary aneurysmal clips for approximately 37 minutes while the tear in the wall of the left vertebral artery was successfully repaired. After arterial repair, extracapsular dissection/resection was undertaken for approximately three hours prior to the tissue being removed en bloc for histological analysis. Histopathology of the surgical specimen confirmed meningioma, which conformed to grade 1 of the World Health Organization (WHO) meningioma grading criteria. Interestingly, there were areas of necrosis seen in several foci spread throughout the entire specimen (Figure 1). However, there was an abundant neutrophil invasion in a characteristic pattern for acute ischemia, as opposed to tumor necrosis seen in high-grade tumors (Figure 2).

## Discussion

While the incidence of meningiomas is disproportionately high when compared to other intracranial tumors, the vast majority (80%) fall into a benign category (grade I) as defined by the World Health Organization (WHO) grading criteria [3]. Grade I indicates a slow growth rate without brain invasion, allowing for gross total resection of the tumor and its dura/bone involvements. Grade II indicates a medium growth rate or brain invasion, and grade III implies a malignant/cancerous tumor. The appropriate means of treatment depends on the grade of meningioma. For instance, while grade I meningiomas are candidates for total resection, higher grades may only allow for partial resection or decompression treatment. As meningiomas grow, they may push against aspects of the vertebrobasilar complex, including the vertebral artery and the anterior inferior cerebellar artery (AICA) [4]. As was the case with our patient, hearing loss and tinnitus may be attributed to neurovascular compression of the vestibulocochlear nerve by the AICA, which may have been pushed forward by the growing tumor identified through radiography [5,6].

Ventral foramen magnum meningiomas account for approximately 3% of all meningiomas and are technically among the most difficult to resect due to their location and close proximity to vital neurovascular structures [7]. Considering this difficulty, the far lateral approach was employed. This approach is well documented for its ability to ease access to meningiomas for surgery in this region [8].

In 1932, Wilder Penfield described meningiomas (the term had been coined by Harvey Cushing 10 years earlier) as having a peripheral vascular supply that is especially sensitive to hypoperfusion, or reduced blood flow, which is a risk factor for developing ischemia. He believed that this central watershed area could lead to ischemic central tumor necrosis and eventual cystic degeneration of meningiomas [2]. This phenomenon has been appreciated histologically in cases of both preoperative embolization and spontaneous infarct of meningiomas. In the case of preoperative embolization, ischemic changes and necrosis are expected histologically, with one study identifying necrosis in 48% and 16% of cases, respectively [9]. The literature also contains several examples of spontaneous infarction of meningiomas presenting with symptoms of rapid neurological decline. In these cases, histopathology of the tumors unsurprisingly revealed necrotic-appearing centers with focal signs of ischemia [10,11]. Past studies have cautioned against the over-grading of preoperatively embolized meningiomas due to the difficulty in distinguishing intrinsic tumor necrosis from necrosis caused by embolization [9,12]. Since the necessity for postoperative radiochemotherapy is determined by the grade of the tumor and residual tumor burden, the accurate grading of such tumors is critical to patient outcomes. In this case, the tumor was not preoperatively embolized, and nor was there any clinical/radiographic evidence to suggest ischemia or infarct prior to surgery.

While the far lateral approach generally involves interfascial dissection to preserve the vertebral artery, its proximity to a proximal branch of meningioma complicated its position during the avulsion of the branch [13]. Inadvertent damage to the vertebral artery during the approach required temporary clipping of the artery proper for circumferential repair, resulting in a total clamp time of 37 minutes. This artery is particularly vulnerable to injury at its V2 and V3 segments. The V2 segment is located within the transverse foramina between C6 and C2, while V3 is located in the transverse foramen of C2 to the cranial dura. As the artery supplies oxygen and nutrients to the spinal cord, cerebellum, medulla, and neck musculature, significant blood loss is a major pitfall [14]. While vertebral artery injuries (VAIs) are rare in cervical spine surgeries (0.2-2%), intra-operative blood loss and hypotension can occur, requiring emergency arterial occlusion and repair, which were performed [15]. Most of the literature describing instances of VAI involves anterior and posterior approaches to the cervical spine, whereas this case illustrates its risk from the far lateral approach. The risk of such a development can be reduced by preoperative imaging to evaluate the position of this artery with regard to the mass being resected.

To the authors’ and pathologist’s great surprise, permanent specimen sent after this time revealed ischemia in a pattern most consistent with acute ischemia with impressive surrounding neutrophils. After the events of acute ischemia, there is an increase in neutrophil production and release from the spleen and bone marrow in combination with a lymphocyte reduction. Neutrophils are the first cells to invade tissue subjected to ischemia, releasing reactive oxygen species, proteases, cytokines, and chemokines, promoting blood clotting (thrombosis) and inflammation [16,17].

To the best of our knowledge, this is the first study of tumoral ischemia resulting from intraoperative maneuvers and, of course, has a great bearing on the histological grading and the subsequent treatment of this tumor. This case illustrates the critical importance of adopting a multidisciplinary approach to complex intracranial pathology, including but not limited to neurosurgery, neurology, radiology, and pathology, in order to provide the best care possible to patients with brain tumors.

## Conclusions

Meningioma was once considered an extremely rare pathological occurrence. However, its incidence has been rising in recent years. This has rendered its pathology to be one of medicinal significance. This case uniquely demonstrated the presence of tumoral ischemia and necrosis resulting from an intraoperative complication, demonstrating the importance of accounting for iatrogenic complications when assessing the appropriate grade of a tumor and its postoperative treatment. It is imperative to seek expertise from such rare outcomes to better aid the medical community in its planning of care for similar patients. The histological analysis coupled with pathological expertise, and a multidisciplinary approach before, during, and after the intervention will reduce such iatrogenic outcomes and decrease the overall risk of suboptimal conditions for the patients.
